# In Vivo View of a Reclining Demodex Mite in a Milia Cyst

**DOI:** 10.3390/diagnostics13101718

**Published:** 2023-05-12

**Authors:** Katharine Hanlon, Meredith Thomley, Lilia Correa-Selm

**Affiliations:** 1 Department of Dermatology and Cutaneous Surgery, University of South Florida, Tampa, FL 33612, USAmereditht@usf.edu (M.T.); 2 Moffitt Cancer Center, Tampa, FL 33612, USA

**Keywords:** confocal microscopy, skin imaging, demodex

## Abstract

Demodex folliculorum and Demodex brevis are commonly present on facial skin and frequently noted via Reflectance Confocal Microscopy (RCM) examination. These mites inhabit follicles and are often seen in groups of two or more, although D. brevis is usually found as a solitary mite. When observed through RCM, they are typically present as refractile, round groupings seen on a transverse image plane inside the sebaceous opening, as they are vertically oriented, and their exoskeletons refract under near-infrared light. Inflammation may occur, leading to a variety of skin disorders; nonetheless, these mites are considered to be part of normal skin flora. a 59-year-old woman presented to our dermatology clinic for confocal imaging (Vivascope 3000, Caliber ID, Rochester, NY, USA) of a previously excised skin cancer for margin evaluation. She did not exhibit symptoms of rosacea or active inflammation of the skin. Incidentally, a solitary demodex mite was noted in a milia cyst nearby the scar. The mite appeared to be trapped in the keratin-filled cyst and was positioned horizontally to the image plane such that its entire body was captured in a coronal orientation as a stack. Demodex identification using RCM can provide clinical diagnostic value in the context of rosacea or inflammation; in our case, this solitary mite was thought to be part of the patient’s normal skin flora. Demodex are practically ubiquitous on the facial skin of older patients and are frequently noted during RCM examination; however, the orientation of the mite referenced herein is uncommon, allowing for a unique view of its anatomy. The use of RCM to identify demodex may become more routine as access to technology grows.

**Figure 1:**
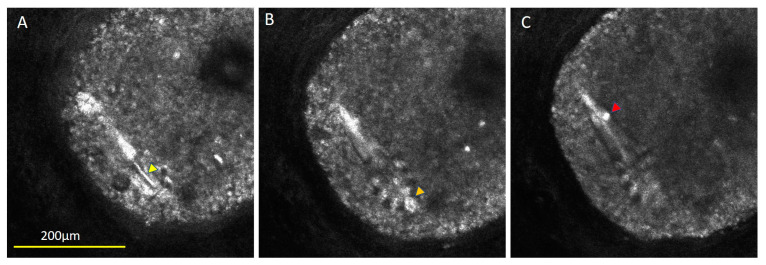
Different aspects of the mite’s anatomy could be visualized as distinct slices. The mite was measured to be 198 µm long and clearly shows 4 pairs of legs, with the gnathasoma having refractile head parts (Panel **B**, orange arrow) and a pointed opisthosoma. These results are associated with a view rarely possible when using RCM due to the typical vertical orientation of the mites when seen in vivo. The pointed opisthosoma and solitary nature of the mite led us to speculate that it was D. brevis [1,2]. Visualization of these body parts from this orientation is sometimes acquired from scanning electron microscopy ex vivo or with light microscopy on a fixed slide, although a few reports of full-body confocal images have been published, wherein the mite was found on the surface of the skin rather than in a follicle [3,4,5]. The RCM image stack of this mite showed some unique findings not evident when using other microscopy approaches. There was a refractile ridge bisecting the podosoma between the leg region (Panel **A**, yellow arrow) and a roundish bright clump seen in the tail region (Panel **C**, red arrow) that could be food or waste. Stacked confocal images can illustrate better the upper, middle, and lower aspects of the mite and its characteristics when visualized in the near-infrared wavelength.

## Data Availability

Original data are unavailable publicly due to patient privacy.
